# Supplementary material for: De novo transcriptome sequence of Senna tora provides insights into anthraquinone biosynthesis
Source: PLoS One. 2020 May 7;15(5):e0225564. doi: 10.1371/journal.pone.0225564 (PMC7205477; doi:10.1371/journal.pone.0225564)
Supplement: S1 Table — (DOCX) [file pone.0225564.s001.docx]

**S1 Table. Gene-specific primers used for tissue-specific qRT-PCR.**

| Unigenes | Annotation | Primer sequence (5’→3’) | Amplicon  (bp) |
| --- | --- | --- | --- |
| c51167_g1F | Cell wall / vacuolar inhibitor of fructosidase 2 | TCTTCATCCCTTTGGTGACA | 135 |
| c51167_g1R |  | ACGATGGTGCAGAGCAGTT |  |
| c97954_g1F | Protein MOTHER of FT and TF 1 | TCACGCATGTTTGGTTCAC | 138 |
| c97954_g1R |  | CACCAATGGCTGCGATATT |  |
| c51098_g1F | Putative glucan 1,3-beta-glucosidase A | CCATTCCAAAGAGCCATCTC | 142 |
| c51098_g1R |  | AAGCGGTACTCTGCAAGCTC |  |
| c30417_g1F | Seed biotin-containing protein SBP65 | CCTCAGTTCCCTGTGGTTG | 149 |
| c30417_g1R |  | CCTCAGTTCCCTGTGGTTG |  |
| c28523_g1F | Poly[ADP-ribose] polymerase 3 | CTCTGCTGTTTCCATCACCA | 112 |
| c28523_g1R |  | CAGCCATTTGGAGTACAACG |  |
| c33220_g1F | Late embryogenesis abundant protein D-29 | CCTGTCCTTAGCCTCCTGTG | 186 |
| c33220_g1R |  | GCTAGTGACATGGCCAACAC |  |
| c49746_g1F | Cytochrome P450 83B1 | TTAGCCTGAAGGCAAAGAGG | 140 |
| c49746_g1R |  | GCATTGGAGCTTATCCTTGC |  |
| c66901_g1F | Receptor-like protein kinase | CCACACCGAATGAGTAGACG | 111 |
| c66901_g1R |  | CGAGGTCCCACATAAGCAC |  |
| c31374_g2F | Peroxisomal (S)-2-hydroxy-acid oxidase | CCGTCCAAGAACACTGGAA | 119 |
| c31374_g2R |  | GCTGGGATAATCGTGTCCA |  |
| c129185_g1F | Organic cation/carnitine transporter 1 | GACAAACCCAACTCCAAACC | 119 |
| c129185_g1R |  | CCACCACAACTGAGGATGAA |  |
| c41007_g1F | WAT1-related protein At1g70260 | GAGTGGGCCCCTGAATAAC | 110 |
| c41007_g1R |  | GCAGGAAGGCAGAGTTGAA |  |
| c22112_g2F | Unknown protein | CTATGAGAGGGATCCGATGG | 116 |
| c22112_g2R |  | GGTGGGAAGCTGTTGTTTG |  |
| c50917_g2F | Internal control (Elongation factor 2) | CCACCATGTTTGGTCCAAGG | 144 |
| c50917_g2R |  | AGGCAATTGATGACGGCAAG |  |
