# Supplementary material for: De novo transcriptome sequence of Senna tora provides insights into anthraquinone biosynthesis
Source: PLoS One. 2020 May 7;15(5):e0225564. doi: 10.1371/journal.pone.0225564 (PMC7205477; doi:10.1371/journal.pone.0225564)
Supplement: S2 Table — (DOCX) [file pone.0225564.s002.docx]

**S2 Table. General properties of the reads produced by Illumina Hiseq 2500 sequencing platform.**

| **Hiseq sequencing library** | **Hiseq2500 reads** | **High-quality reads rate (>=Q30) (%)** | **Mapping rate**  **(%)** |
| --- | --- | --- | --- |
| Leaf_1 | 19,606,595 | 88.71 | 81.91 |
| Leaf_2 | 19,605,925 | 89.40 | 82.16 |
| Leaf_3 | 20,355,521 | 92.52 | 85.70 |
| Root_1 | 22,299,323 | 92.33 | 83.99 |
| Root_2 | 21,073,842 | 92.33 | 85.34 |
| Root_3 | 22,322,536 | 92.50 | 85.37 |
| Early Seed_1 | 24,629,055 | 88.82 | 82.45 |
| Early Seed_2 | 24,794,874 | 88.62 | 82.69 |
| Early Seed_3 | 30,257,044 | 88.61 | 81.14 |
| Late Seed_1 | 25,197,545 | 92.28 | 86.40 |
| Late Seed_2 | 24,216,679 | 92.08 | 86.83 |
| Late Seed_3 | 23,672,556 | 88.15 | 81.56 |
