# Supplementary material for: De novo transcriptome sequence of Senna tora provides insights into anthraquinone biosynthesis
Source: PLoS One. 2020 May 7;15(5):e0225564. doi: 10.1371/journal.pone.0225564 (PMC7205477; doi:10.1371/journal.pone.0225564)
Supplement: S3 Table — (DOCX) [file pone.0225564.s003.docx]

**S3 Table. General properties of the reads produced by PacBio sequencing platform.**

| **PacBio sequencing library** | **PacBio reads** | **Full-length reads** | **High-quality isoforms** |
| --- | --- | --- | --- |
| <2 kb | 198,919 | 96,228 | 39,672 |
| 2-3 kb | 202,789 | 101,612 | 32,954 |
| >3 kb | 367,037 | 187,029 | 46,077 |
