# Supplementary material for: De novo transcriptome sequence of Senna tora provides insights into anthraquinone biosynthesis
Source: PLoS One. 2020 May 7;15(5):e0225564. doi: 10.1371/journal.pone.0225564 (PMC7205477; doi:10.1371/journal.pone.0225564)
Supplement: S4 Table — (DOCX) [file pone.0225564.s004.docx]

**S4 Table. Tissue-enriched and specific transcription factors (TFs) distribution of each tissue.**

|  | **Leaf** | | | **Root** | | | **Early Seed** | | | **Late Seed** | | |
| --- | --- | --- | --- | --- | --- | --- | --- | --- | --- | --- | --- | --- |
| **TF**  **Family** | **Tissue**  **Enriched** | **Tissue**  **Specific** | **No.** | **Tissue**  **Enriched** | **Tissue**  **Specific** | **No.** | **Tissue**  **Enriched** | **Tissue**  **Specific** | **No.** | **Tissue**  **Enriched** | **Tissue**  **Specific** | **No.** |
| AP2 | 0 | 1 | 1 | 1 | 0 | 1 | 0 | 1 | 1 | 1 | 0 | 1 |
| ARF | 0 | 1 | 1 | 4 | 0 | 4 | 0 | 0 | 0 | 0 | 0 | 0 |
| ARR-B | 1 | 0 | 1 | 0 | 0 | 0 | 0 | 0 | 0 | 0 | 0 | 0 |
| B3 | 0 | 0 | 0 | 0 | 0 | 0 | 1 | 1 | 2 | 2 | 0 | 2 |
| BES1 | 0 | 3 | 3 | 0 | 0 | 0 | 0 | 0 | 0 | 0 | 0 | 0 |
| bHLH | 5 | 9 | 14 | 1 | 13 | 14 | 4 | 9 | 13 | 2 | 1 | 3 |
| bZIP | 0 | 3 | 3 | 3 | 7 | 10 | 3 | 7 | 10 | 0 | 1 | 1 |
| C2H2 | 1 | 2 | 3 | 4 | 15 | 19 | 2 | 3 | 5 | 0 | 0 | 0 |
| C3H | 2 | 1 | 3 | 0 | 1 | 1 | 1 | 1 | 2 | 1 | 2 | 3 |
| CO-like | 1 | 2 | 3 | 0 | 0 | 0 | 0 | 0 | 0 | 0 | 0 | 0 |
| CPP | 0 | 0 | 0 | 0 | 0 | 0 | 1 | 0 | 1 | 0 | 0 | 0 |
| Dof | 0 | 2 | 2 | 0 | 1 | 1 | 2 | 1 | 3 | 0 | 0 | 0 |
| ERF | 6 | 6 | 12 | 1 | 1 | 2 | 0 | 3 | 3 | 1 | 3 | 4 |
| FAR1 | 0 | 0 | 0 | 0 | 0 | 0 | 1 | 0 | 1 | 0 | 0 | 0 |
| G2-like | 1 | 4 | 5 | 0 | 5 | 5 | 0 | 0 | 0 | 0 | 0 | 0 |
| GATA | 0 | 0 | 0 | 0 | 0 | 0 | 2 | 0 | 2 | 0 | 3 | 3 |
| GeBP | 0 | 0 | 0 | 0 | 0 | 0 | 0 | 0 | 0 | 0 | 0 | 0 |
| GRAS | 1 | 0 | 1 | 2 | 0 | 2 | 0 | 1 | 1 | 1 | 0 | 1 |
| GRF | 0 | 0 | 0 | 0 | 0 | 0 | 0 | 0 | 0 | 3 | 2 | 5 |
| HD-ZIP | 0 | 1 | 1 | 2 | 3 | 5 | 1 | 0 | 1 | 0 | 0 | 0 |
| HSF | 0 | 0 | 0 | 0 | 3 | 3 | 0 | 0 | 0 | 0 | 2 | 2 |
| LBD | 1 | 0 | 1 | 0 | 2 | 2 | 1 | 2 | 3 | 0 | 1 | 1 |
| LFY | 0 | 0 | 0 | 0 | 0 | 0 | 0 | 0 | 0 | 0 | 1 | 1 |
| LSD | 1 | 0 | 1 | 0 | 0 | 0 | 1 | 0 | 1 | 0 | 0 | 0 |
| MIKC | 2 | 2 | 4 | 3 | 9 | 12 | 3 | 8 | 11 | 0 | 0 | 0 |
| M-type | 0 | 0 | 0 | 0 | 1 | 1 | 0 | 0 | 0 | 0 | 0 | 0 |
| MYB | 0 | 8 | 8 | 8 | 9 | 17 | 2 | 6 | 8 | 2 | 1 | 3 |
| MYB-related | 1 | 3 | 4 | 0 | 5 | 5 | 0 | 1 | 1 | 0 | 0 | 0 |
| NAC | 3 | 7 | 10 | 4 | 8 | 12 | 2 | 0 | 2 | 0 | 0 | 0 |
| NF-YA | 0 | 0 | 0 | 1 | 1 | 2 | 0 | 0 | 0 | 0 | 0 | 0 |
| NF-YB | 0 | 0 | 0 | 0 | 1 | 1 | 0 | 2 | 2 | 0 | 0 | 0 |
| Nin-like | 0 | 0 | 0 | 0 | 1 | 1 | 0 | 0 | 0 | 1 | 0 | 1 |
| RAV | 0 | 2 | 2 | 0 | 0 | 0 | 0 | 0 | 0 | 0 | 0 | 0 |
| SBP | 1 | 0 | 1 | 0 | 0 | 0 | 0 | 1 | 1 | 0 | 0 | 0 |
| SRS | 0 | 0 | 0 | 0 | 1 | 1 | 0 | 0 | 0 | 0 | 0 | 0 |
| TALE | 0 | 5 | 5 | 2 | 3 | 5 | 0 | 0 | 0 | 0 | 0 | 0 |
| TCP | 0 | 2 | 2 | 0 | 0 | 0 | 0 | 0 | 0 | 0 | 1 | 1 |
| Trihelix | 0 | 2 | 2 | 0 | 2 | 2 | 0 | 2 | 2 | 0 | 0 | 0 |
| WOX | 0 | 0 | 0 | 0 | 0 | 0 | 0 | 1 | 1 | 1 | 0 | 1 |
| WRKY | 8 | 32 | 40 | 5 | 3 | 8 | 0 | 0 | 0 | 0 | 0 | 0 |
| YABBY | 0 | 0 | 0 | 0 | 0 | 0 | 3 | 1 | 4 | 0 | 0 | 0 |
| ZF-HD | 0 | 0 | 0 | 0 | 2 | 2 | 0 | 0 | 0 | 0 | 0 | 0 |
| Total | 35 | 98 | 133 | 41 | 97 | 138 | 30 | 51 | 81 | 15 | 18 | 33 |
