# Supplementary material for: De novo transcriptome sequence of Senna tora provides insights into anthraquinone biosynthesis
Source: PLoS One. 2020 May 7;15(5):e0225564. doi: 10.1371/journal.pone.0225564 (PMC7205477; doi:10.1371/journal.pone.0225564)
Supplement: S5 Table — (DOCX) [file pone.0225564.s005.docx]

**S5 Table. Gene associated with the secondary metabolite pathway in *S. tora***

| **Pathway** | **Gene** | **Symbol** | **RNA-Seq** | **Iso-Seq** |
| --- | --- | --- | --- | --- |
| MEP/DOXP Pathway | 1-Deoxy-_D_-xylulose-5-phosphate synthase (EC 2.2.1.7) | DXPS | 13 | 10 |
|  | 1-Deoxy-_D_-xylulose-5-phosphate reductoisomerase (EC 1.1.1.267) | DXR | 4 | 3 |
|  | 2-C-Methyl-_D_-erythritol 4-phosphate cytidylyltransferase (EC 2.7.7.60) | ISPD | 1 | 2 |
|  | 4-Diphosphocytidyl-2-C-methyl-_D_-erythritol kinase (EC 2.7.1.148) | CDPMEK | 1 | 1 |
|  | 2-C-Methyl-_D_-erythritol 2,4-cyclodiphosphate Synthase (EC 4.6.1.12) | ISPF | 2 | 1 |
|  | (E)-4-Hydroxy-3-methylbut-2-enyl-diphosphate synthase (EC 1.17.7.1) | HDS | 8 | 3 |
|  | 4-Hydroxy-3-methylbut-2-enyl diphosphate reductase (EC 1.17.1.2) | HDR | 6 | 4 |
| MEV  Pathway | Acetyl-CoA carboxylase (EC 6.4.1.2) | ACCA | 29 | 9 |
|  | Hydroxymethylglutaryl-CoA synthase (EC 2.3.3.10) | HMGS | 4 | 5 |
|  | Hydroxymethylglutaryl-CoA reductase (EC 1.1.1.34) | HMGR | 9 | 3 |
|  | Mevalonate kinase (EC 2.7.1.36) | MK | 5 | 2 |
|  | Phosphomevalonate kinase (EC 2.7.4.2) | PMK | 3 | 0 |
|  | Methyl parathion hydrolase (EC 3.1.8.1) | MPD | 1 | 1 |
|  | Isopentenyl-diphosphate delta-isomerase (EC 5.3.3.2) | IPPS | 4 | 2 |
| Shikimate Pathway | 3-Deoxy-7-phosphoheptulonate synthase (EC:2.5.1.54) | DAHPS | 11 | 7 |
|  | 3-Dehydroquinate synthase (EC:4.2.3.4) | DHQS | 2 | 2 |
|  | 3-Dehydroquinate dehydratase/shikimate dehydrogenase  (EC 4.2.1.10/1.1.1.25) | DHQD/SDH | 2 | 3 |
|  | Shikimate kinase (EC:2.7.1.71) | SMK | 8 | 4 |
|  | 3-Phosphoshikimate 1-carboxyvinyltransferase (EC:2.5.1.19) | EPSP | 7 | 3 |
|  | Chorismate synthase (EC:4.2.3.5) | CS | 5 | 1 |
|  | Isochorismate synthase (EC 5.4.4.2) | ICS | 2 | 2 |
|  | 2-Succinylbenzoate--CoA ligase (EC 6.2.1.26) | MenE | 1 | 6 |
|  | 1,4-Dihydroxy-2-naphthoyl-CoA synthase (EC 4.1.3.36) | MenB | 2 | 3 |
| Carotenoid Pathway | Geranylgeranyl diphosphate synthase (EC 2.5.1.1) | GGPS | 15 | 4 |
|  | Phytoene synthase (EC 2.5.1.32) | PSY | 9 | 5 |
|  | Phytoene desaturase (EC 1.3.99.30) | PDS | 2 | 4 |
|  | Zeta-carotene desaturase (EC 1.3.5.6 | ZDS | 3 | 3 |
|  | Lycopene beta-cyclase (EC 5.5.1.19) | LYCB | 3 | 1 |
|  | Lycopene epsilon-cyclase (EC 5.5.1.18) | LYCE | 1 | 1 |
|  | Beta-carotene hydroxylase (EC 1.14.13.129) | BCH | 3 | 3 |
|  | Zeaxanthin epoxidase (EC 1.14.15.21) | ZEP | 7 | 13 |
| Flavonoid/  Polyketide Pathway | Phenylalanine ammonia-lyase (EC 4.3.1.24) | PAL | 17 | 7 |
|  | Cinnamate-4-hydroxylase (EC 1.14.13.11) | C4H | 3 | 1 |
|  | 4-Coumarate--CoA ligase (EC 6.2.1.12) | 4CL | 32 | 16 |
|  | Chalcone synthase (EC 2.3.1.74) | CHS/PKS | 27 | 23 |
|  | Chalcone isomerase (EC 5.5.1.6) | CHI | 4 | 0 |
|  | Polyketide cyclase | PKC | 1 | 2 |
|  | Flavanone 3-hydroxylase/Flavonol synthase (EC 1.14.11.9) | F3H | 10 | 6 |
|  | Dihydrokaempferol 4-reductase (EC 1.1.1.219) | DFR | 5 | 1 |
|  | Leucoanthocyanidin dioxygenase (EC 1.14.20.4) | LDOX | 7 | 0 |
|  | UDP-glucosyltransferase (EC 2.4.1.17) | UGT | 59 | 47 |
