# Supplementary figures and images for: De novo transcriptome sequence of Senna tora provides insights into anthraquinone biosynthesis
Source: PLoS One. 2020 May 7;15(5):e0225564. doi: 10.1371/journal.pone.0225564 (PMC7205477; doi:10.1371/journal.pone.0225564)

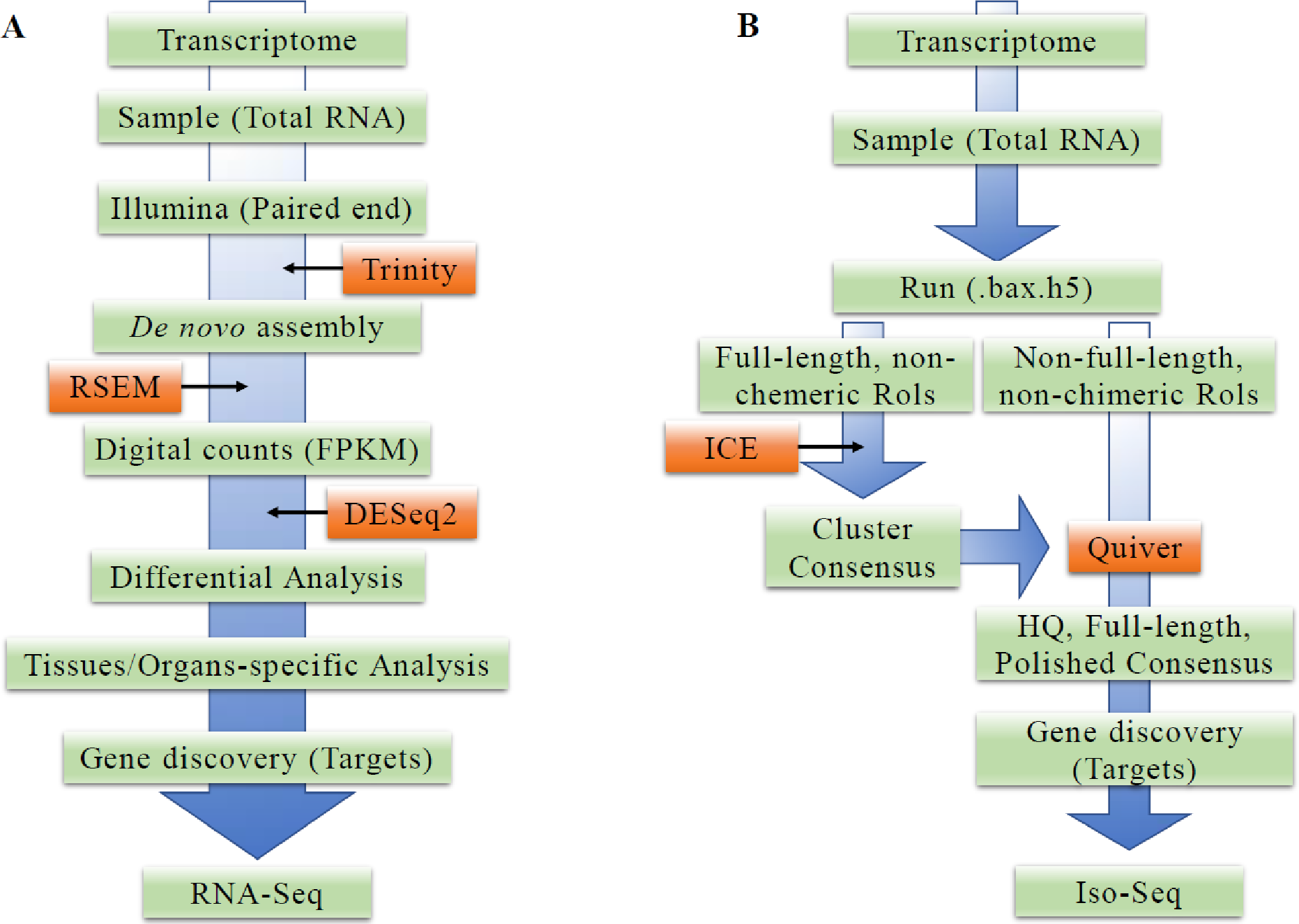

Supplement: S1 Fig — Overview of analysis workflow for RNA-Seq (A) and Iso-Seq (B) transcriptional profiling. (TIF) [file pone.0225564.s006.tif]

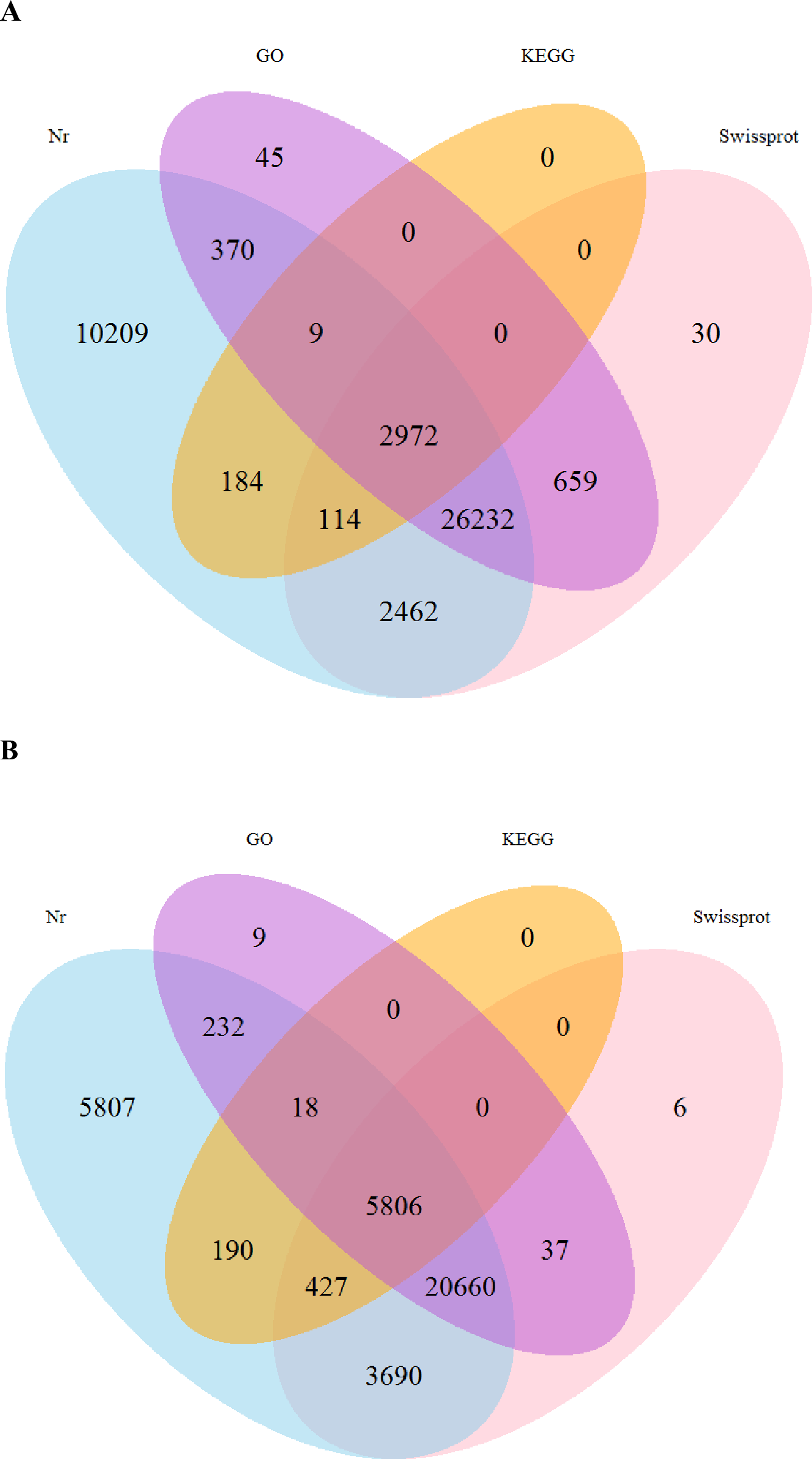

Supplement: S2 Fig — Venn diagram showing the proportion of annotated unigenes in NCBI Nr, KEGG, Swiss-Prot, and GO databases with RNA-Seq (A) and Iso-Seq (B). (TIF) [file pone.0225564.s007.tif]

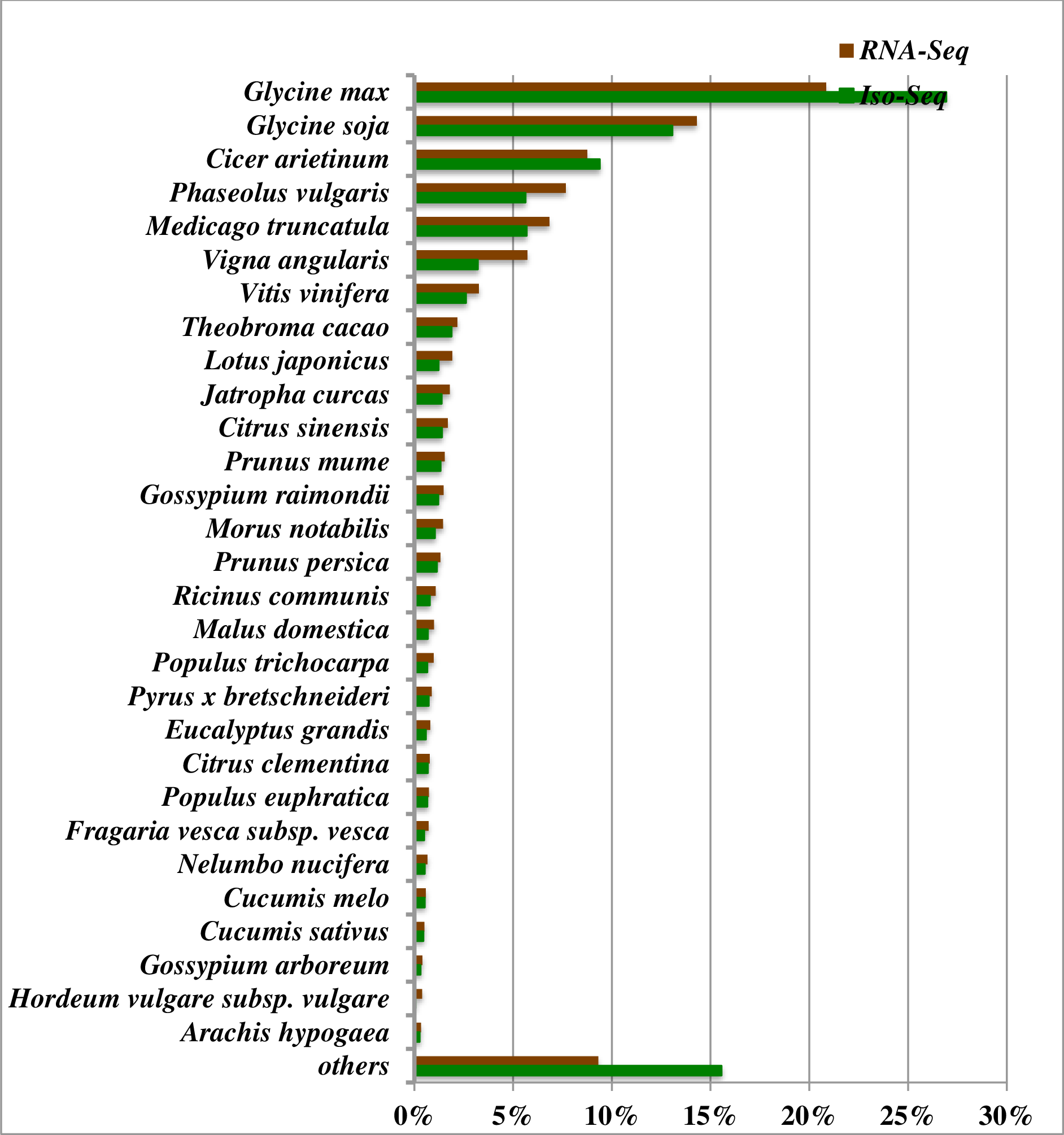

Supplement: S3 Fig — Top-hit species from RNA-Seq and Iso-Seq were calculated based on sequence alignments with the lowest E-value obtained from BLAST. (TIF) [file pone.0225564.s008.tif]

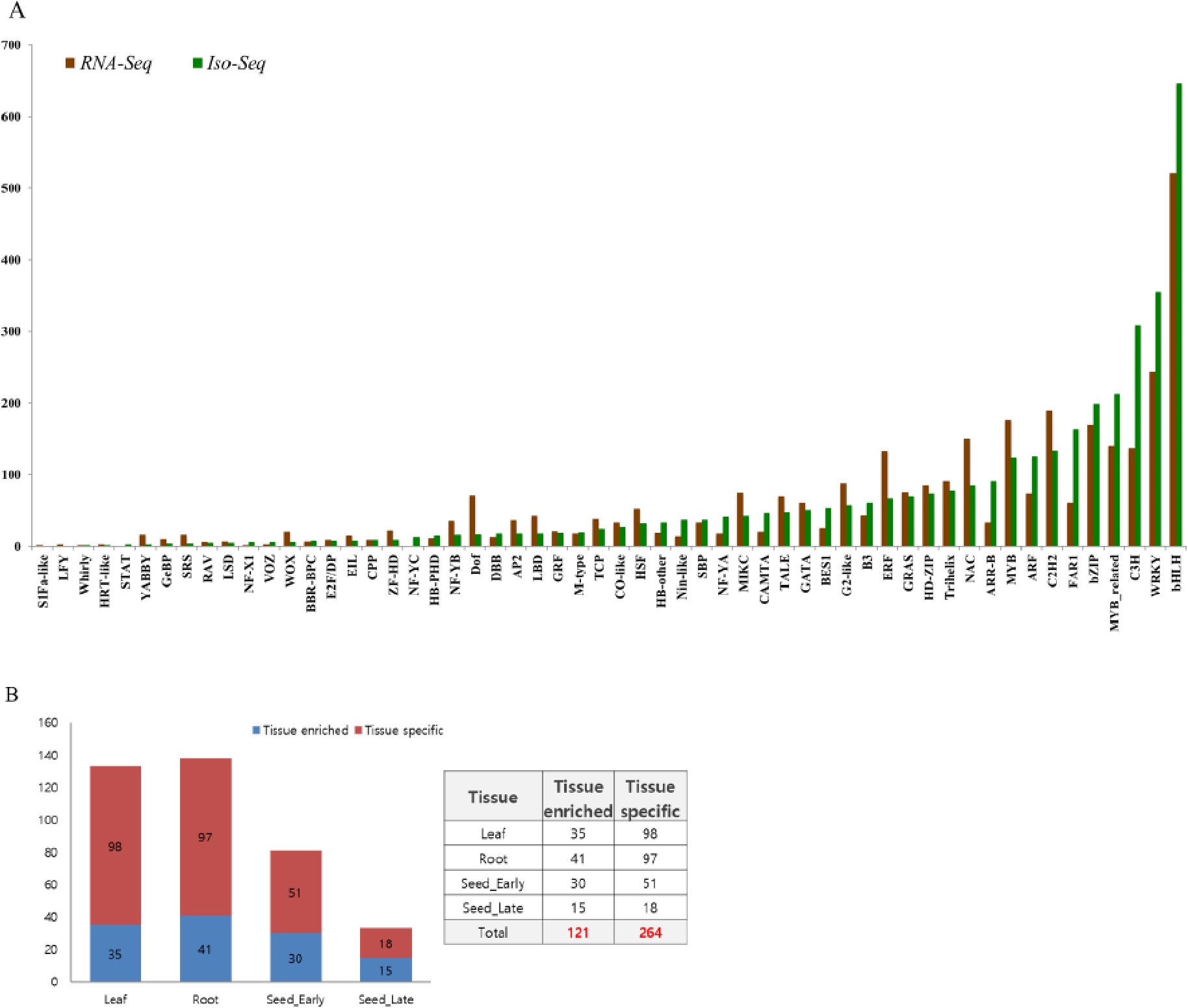

Supplement: S4 Fig — Distribution of transcripts (3,284 for RNA-Seq and 3,576 for Iso-Seq) that encode for transcription factors (A). Number of transcripts exhibiting specific expression in different tissues has been indicated by bar and table (B). Tissue-specific shows 10-fold higher FPKM in one tissue compared with three tissues, and tissue-enriched represents 5-fold higher FPKM compared with other tissues. (TIF) [file pone.0225564.s009.tif]

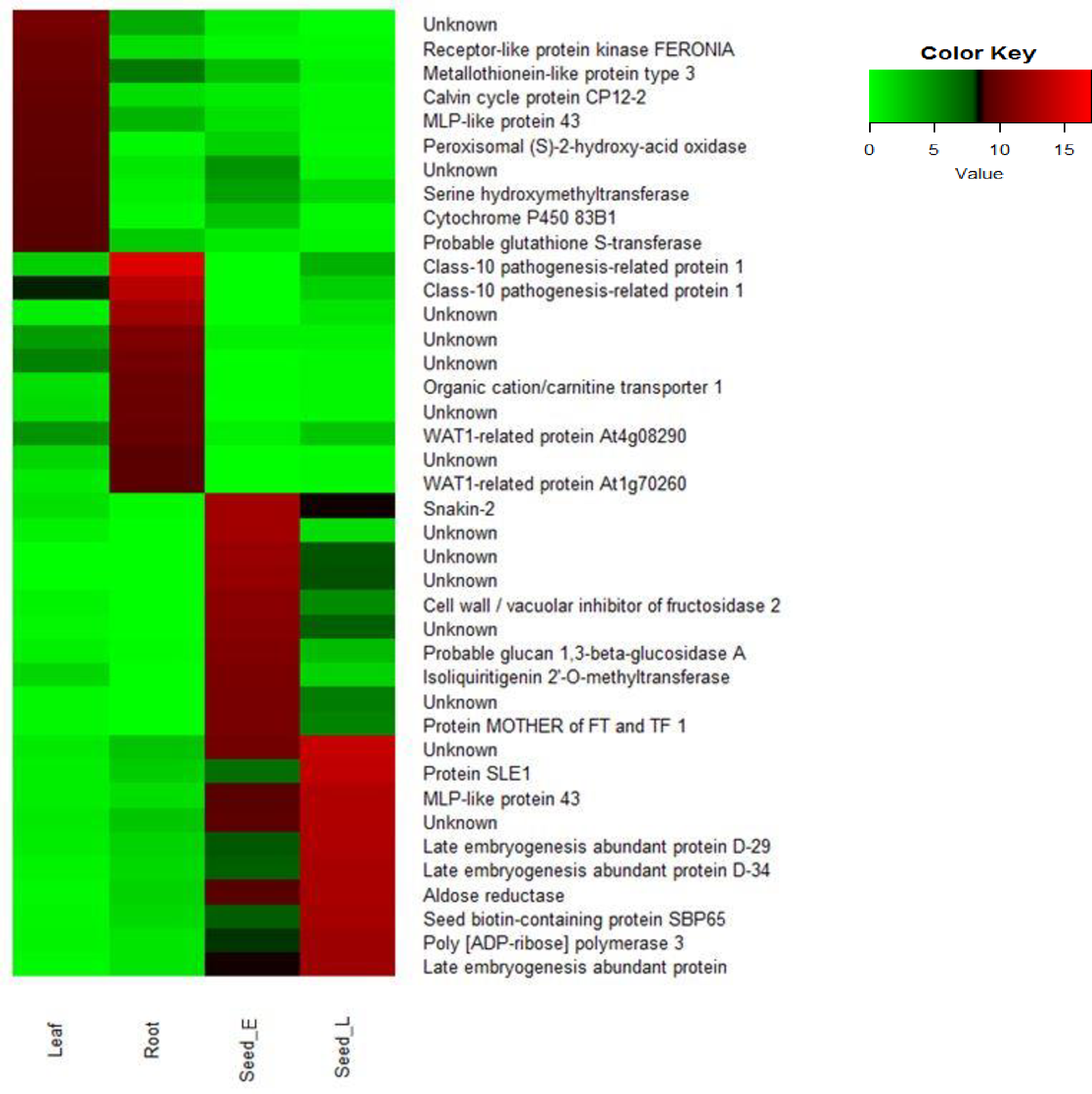

Supplement: S5 Fig — Red represents high abundance and green represents low abundance. (TIF) [file pone.0225564.s010.tif]

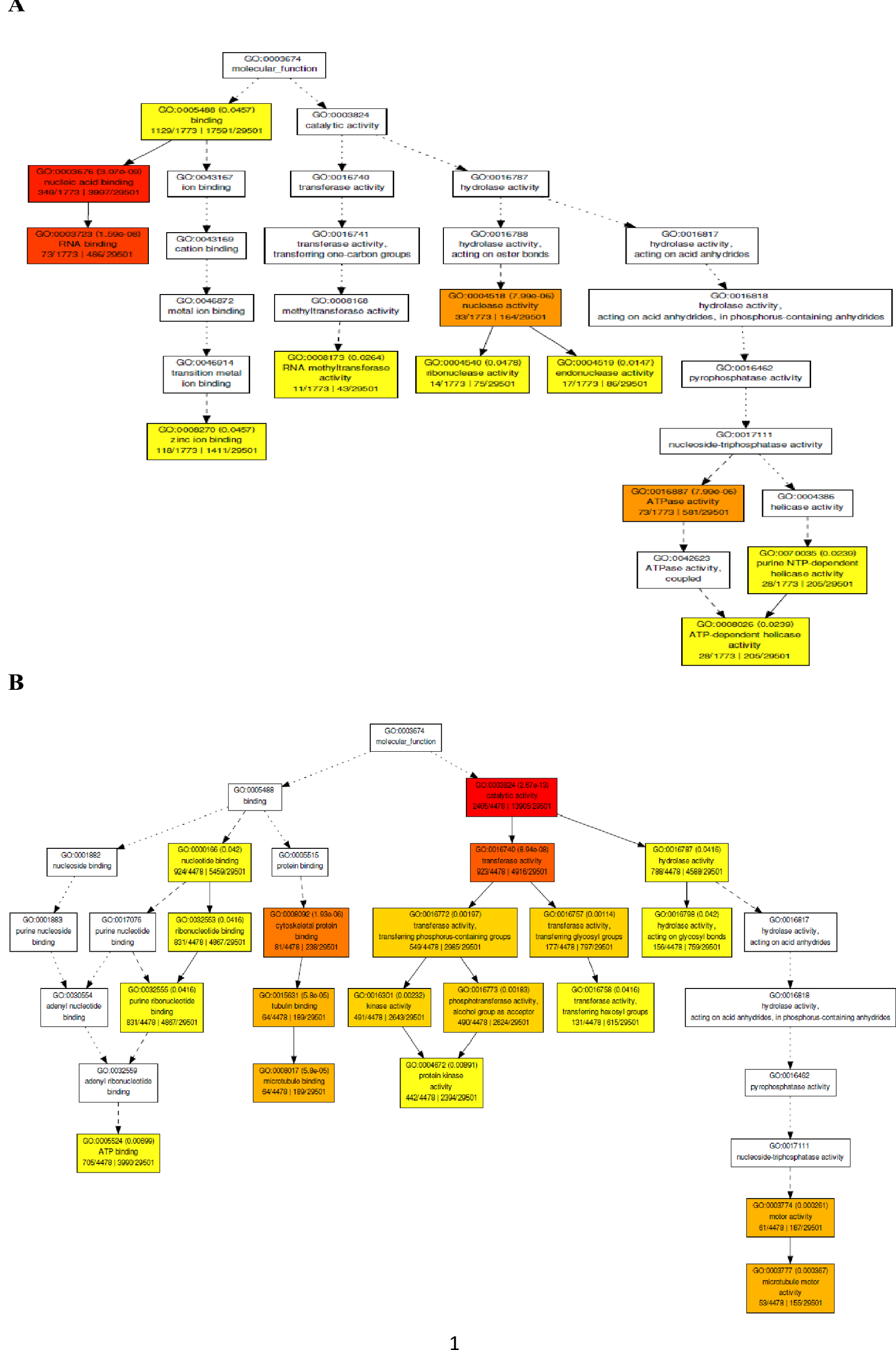

Supplement: S6 Fig — A total of 4,935 (up-regulated, A) and 9,890 (down-regulated, B) genes with Molecular terms are represented by increasingly red colors. GO term enrichment was performed using single enrichment analysis (SEA) tool on AgriGo (http://bioinfo.cau.edu.cn/agrigo/). Box colors indicates levels of statistical significance: yellow = 0.05; orange = e-05; and red = e-09. (TIF) [file pone.0225564.s011.tif]
